# Regulating and Cultural Ecosystem Services of Urban Green Infrastructure in the Nordic Countries: A Systematic Review

**DOI:** 10.3390/ijerph18031219

**Published:** 2021-01-29

**Authors:** Jorge H. Amorim, Magnuz Engardt, Christer Johansson, Isabel Ribeiro, Magnus Sannebro

**Affiliations:** 1 Swedish Meteorological and Hydrological Institute (SMHI), 601 76 Norrköping, Sweden; isabel.ribeiro@smhi.se; 2 Environment and Health Administration, Box 8136, 104 20 Stockholm, Sweden; magnuz.engardt@slb.nu (M.E.); christer.johansson@slb.nu (C.J.); magnus.sannebro@stockholm.se (M.S.); 3 Department of Environmental Science, Stockholm University, 106 91 Stockholm, Sweden

**Keywords:** urban green infrastructure, ecosystem services, Nordic countries, urban climate, heat, flood, air pollution, well-being, health, end users

## Abstract

In the Nordic countries (Denmark, Finland, Iceland, Norway and Sweden), the Urban Green Infrastructure (UGI) has been traditionally targeted at reducing flood risk. However, other Ecosystem Services (ES) became increasingly relevant in response to the challenges of urbanization and climate change. In total, 90 scientific articles addressing ES considered crucial contributions to the quality of life in cities are reviewed. These are classified as (1) regulating ES that minimize hazards such as heat, floods, air pollution and noise, and (2) cultural ES that promote well-being and health. We conclude that the planning and design of UGI should balance both the provision of ES and their side effects and disservices, aspects that seem to have been only marginally investigated. Climate-sensitive planning practices are critical to guarantee that seasonal climate variability is accounted for at high-latitude regions. Nevertheless, diverging and seemingly inconsistent findings, together with gaps in the understanding of long-term effects, create obstacles for practitioners. Additionally, the limited involvement of end users points to a need of better engagement and communication, which in overall call for more collaborative research. Close relationships and interactions among different ES provided by urban greenery were found, yet few studies attempted an integrated evaluation. We argue that promoting interdisciplinary studies is fundamental to attain a holistic understanding of how plant traits affect the resulting ES; of the synergies between biophysical, physiological and psychological processes; and of the potential disservices of UGI, specifically in Nordic cities.

## 1. Introduction

Studies from all across the globe have shown that the Urban Green Infrastructure (UGI) holds the potential to increase the urban resilience to climate change and to multiple hazards, from floods and heat waves to anthropogenic air pollution and noise. These benefits have been commonly known as Ecosystem Services (ES). UGI offers also more sustainable solutions for water management and food production, among other natural amenities that promote both biodiversity and the life quality of urban dwellers (e.g., [1,2,3,4,5,6,7,8]).

Despite efforts to include these ES in urban management [9], cities have received less attention than other ecosystems, for example wetlands and forests [10], in major international initiatives for the valuation of ES [11,12,13,14]. Some benefits that are relevant in the urban context, such as health-related ones, are therefore not well captured and represented in common ES classification systems [14], notwithstanding recent attempts [6]. Moreover, city specificities, namely climate, physiography and population, add an extra complexity to the valuation of ES.

In the Nordic countries (Denmark, Finland, Iceland, Norway and Sweden), the UGI has been traditionally targeted at reducing stormwater peak flow and runoff volume during heavy rainfall [15]. However, the record hot summer of 2018 in Scandinavia, and projections of 2–4 °C increased temperatures of the hottest days in Northern Europe [16], have raised the awareness of the potential health effects of heat waves, stimulating the interest for the capacity of nature-based solutions to reduce heat stress. In parallel, these hazards may be amplified by ongoing urbanization (e.g., [17]) due to changes in the form and function of the landscape (see [18] for more details). Moreover, urban growth poses additional societal, economic and environmental risks (e.g., [19]) that will ultimately impact the well-being and health of the population (see, e.g., Figures A and B in [11]).

While the growing interest for the ES provided by urban nature has motivated a plethora of studies (e.g., [1,2,3,4,5,6,7,8]), there is a lack of a comprehensive retrieval, synthetization and appraisal of the existing knowledge on the role of UGI specifically for the conditions of Nordic cities. Furthermore, although holistic evaluations of both the services and disservices of UGI have been proposed [3], the latter are frequently neglected or less well understood, which may compromise the implementation of UGI solutions by end users [15]. Examples include the build-up of traffic-related air pollution hot-spots in street canyons due to reduced ventilation [20]), or the exclusion of pollen grains allergenicity as a selection criterion in urban planning [3]).

In this context, our study evaluates the current state of knowledge and identifies gaps on (1) the regulation of climate, urban heat, floods, air pollution and noise, and on (2) the promotion of well-being and health, commonly designated as cultural ES, specifically in the Nordic countries. These ES are considered crucial contributions to the quality of life in cities [10], justifying the topic choice for this review. In particular, we aim to answer the following research questions:

(1) In addition to flood regulation, have other ES of UGI emerged in the research panorama across the Nordic countries?
(2) Is there a solid scientific evidence of the (regulating/cultural) benefits commonly attributed to UGI?
(3) Is there an equivalent understanding of the potential co-benefits and disservices?
(4) Are there scientific gaps that may hinder the implementation of UGI solutions in this region?

## 2. Methods

**Figure 1:**
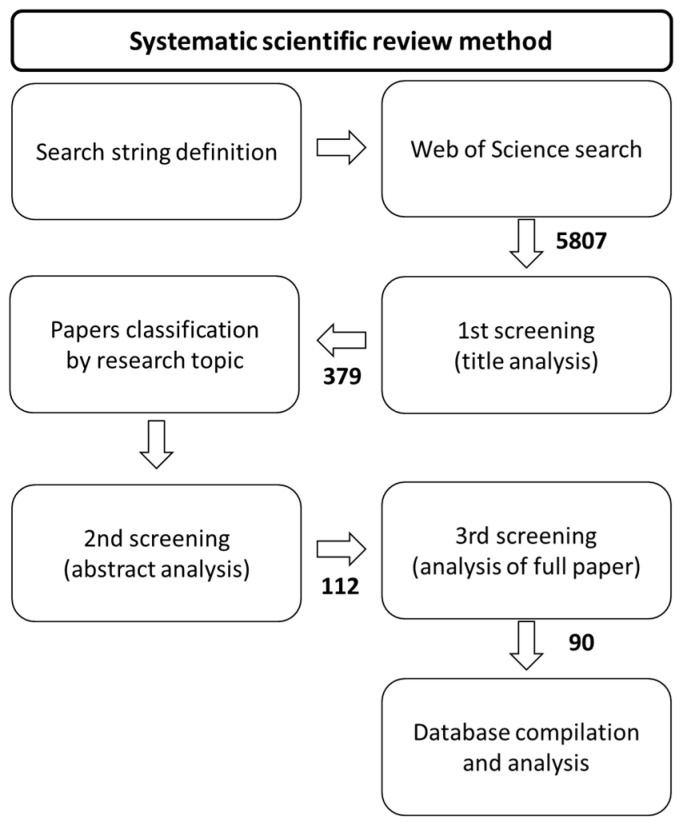
Main stages of the systematic review indicating the number of scientific papers processed on each stage. When unclear, papers were conditionally moved forward.

The systematic review schematically shown in Figure 1 considered peer-reviewed articles published in English from January 2008 to March 2018 in scientific journals indexed to Web of Science Core Collection, counting at the date a total of 20.5 million records. The eligibility criteria consisted of (1) studies conducted in cities of Denmark, Finland, Iceland, Norway and Sweden; (2) including any form of urban vegetation and plant species; and (3) addressing regulating and/or cultural ES according to the classification and definitions adapted from [11,12,14]. Aiming to limit the scope of the analysis, we have deliberately restricted the use of medical and epidemiological terminology in the search string (see Table A1 in Appendix A). Relevant metadata was compiled for each article using the database structure shown in Table A2.

## 3. Results

**Figure 2:**
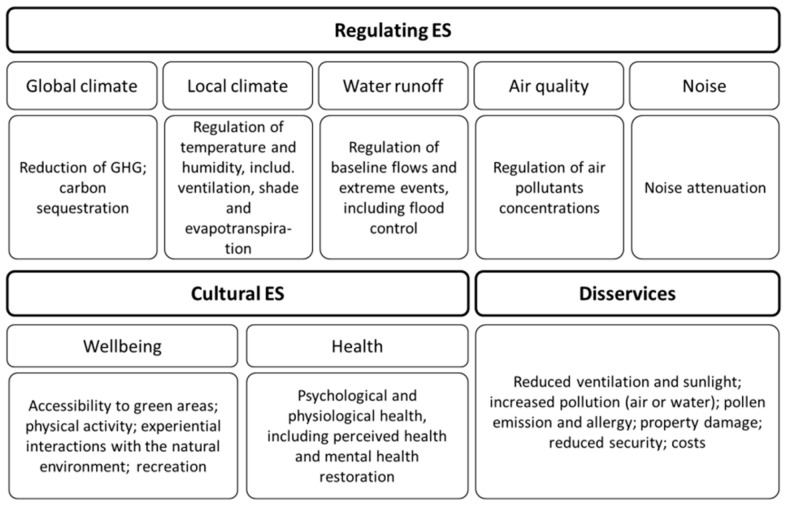
Main Ecosystem Services (ES) and disservices, their processes and effects covered by the review. ES classification adapted from [11,12,14].

From the process described in Figure 1, 90 scientific articles were selected for full review. These were then distributed into regulating and cultural ES following the classification presented in Figure 2. Negative impacts, known as disservices, were also compiled and analyzed.

### 3.1. Metadata

#### 3.1.1. Research Topic

**Figure 3:**
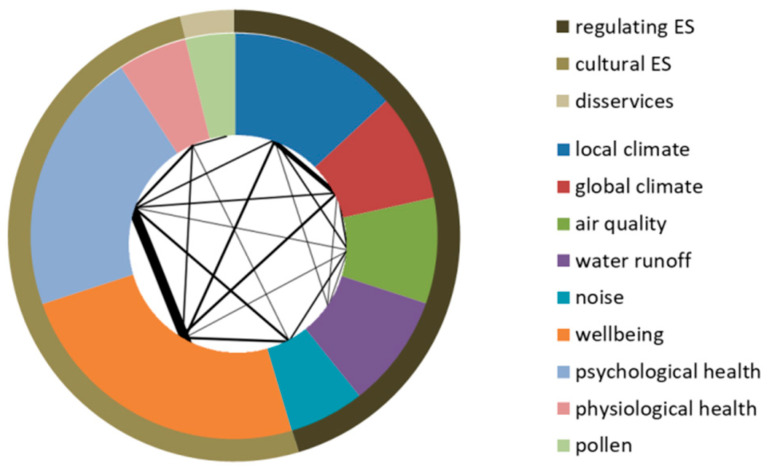
Distribution of articles by ES (outer circle) and topic (inner circle). The spider links topics addressed simultaneously, while the line thickness is proportional to the number of articles. The complete list of papers reviewed is given in Table A3.

Topic wise, regulating and cultural ES are addressed in a similar number of articles (Figure 3). Nearly half investigate more than one topic, in some cases multiple ES are valued with the objective of supporting routine planning processes (e.g., [21]). The spider diagram reveals the emphasis on the effects of green spaces on wellbeing and psychological health (cultural ES), as also towards the regulation of local and global climate. The emission of pollen is the disservice most frequently addressed in Nordic cities.

#### 3.1.2. UGI Type and Research Method

**Figure 4:**
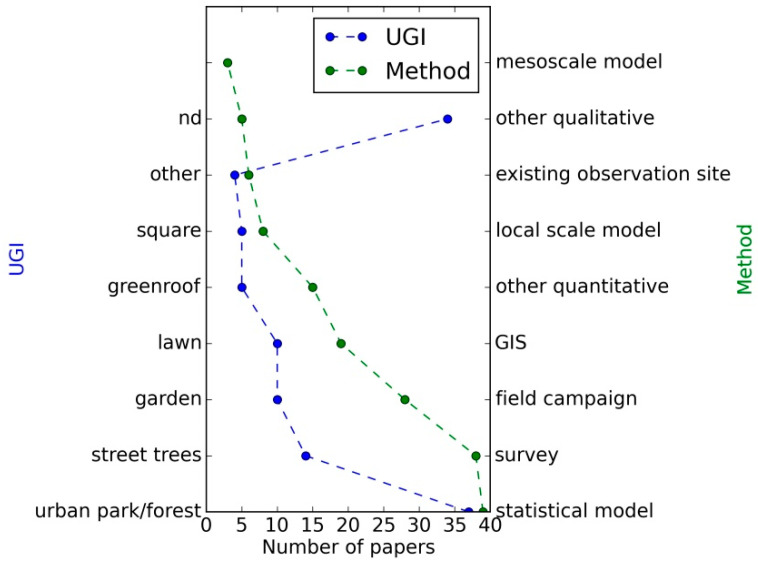
Distribution of number of articles by Urban Green Infrastructure (UGI) type and research method. “nd” stands for unspecified or undetermined (e.g., green spaces).

Figure 4 identifies a clear focus on urban parks and forests, which despite morphological and functional differences are often treated indistinctively in the literature. Street trees are also common, being mostly included in small-scale heat-related studies, while the use of allotment gardens expresses a distinguishing characteristic of Nordic cities. Green roofs have a relevant role within flood control management.

An extensive number of surveys and interviews, processed with statistical and GIS (Geographical Information System) tools, have collected the people’s perceptions of well-being in relation to nature experiences. Measurement campaigns, complemented by observations from existing sites, are more frequently used in the analysis of regulating ES than numerical modelling, either at local or regional scales, while GIS methods are common in the analysis of nature accessibility and ES valuation.

#### 3.1.3. Spatiotemporal Scales of the Analysis

**Figure 5:**
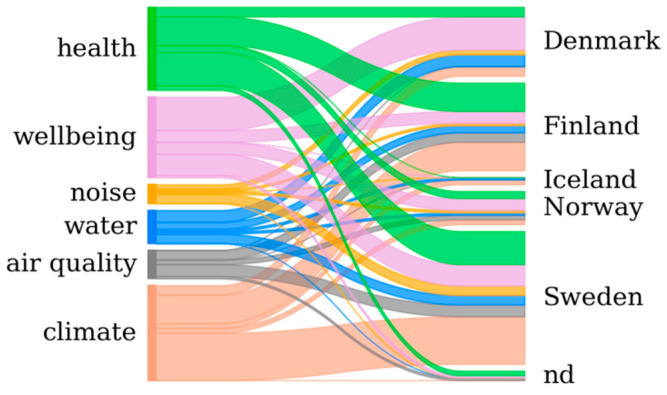
Distribution of research topics by country (note that papers with a multi-country analysis are counted accordingly). “nd” stands for undetermined (e.g., Northern Europe or Scandinavia) or unspecified.

The largest fraction of works (38%) in our assessment have been focused in Swedish cities, notably on local climate and psychological health, as depicted in Figure 5. Benefits to well-being or in the control of floods have driven substantial research in Denmark, while in Finland the focus lies on climate and health. Expectably, few publications address Icelandic cities, but the representativeness of Norwegian works is proportionally low. The country capital dominates as preferred research location (Helsinki 87%, Copenhagen and Oslo 67%), with the exception of Sweden where Gothenburg is more frequently addressed (50%, against 23% for Stockholm).

**Figure 6:**
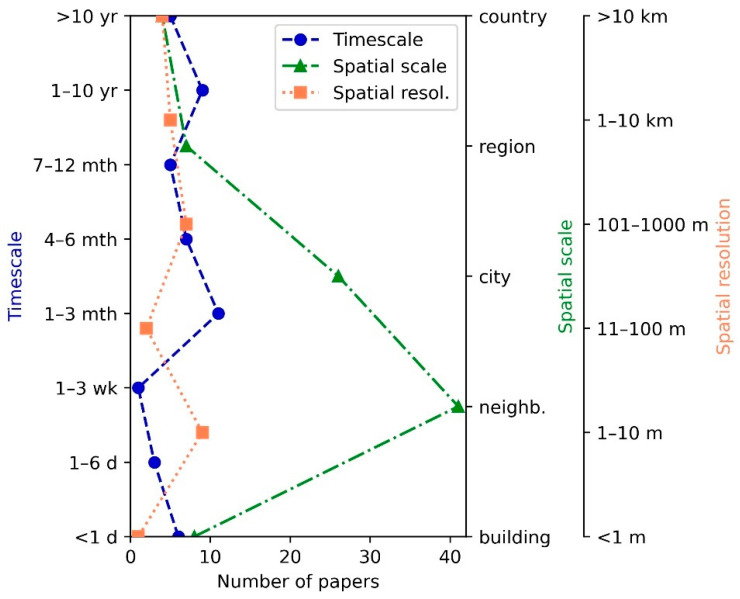
Spatial scale/resolution and time scale. The spatial resolution applies to modelling studies or spatial analyses (e.g., GIS works using raster data), where information about grid cell size is available. The time scale indicates the duration of the research (e.g., the period covered by a monitoring campaign or a model study). (d: day, wk: week, mth: month, yr: year, neighb.: neighborhood).

A prevalence of studies at the scale of the neighborhood (e.g., parks and forests) and the city is clear in Figure 6. The spatial resolution, which applies predominantly to modelling studies, varies typically between 1 and 10 m or above 100 m, with very high resolution (below 1 m in grid size) being almost absent in our review. There is a tendency for research, especially in air quality, to take between 1 and 3 months.

### 3.2. Ecosystem Services Provision

#### 3.2.1. Climate Regulation

Long-term direct flux measurements in Helsinki using the eddy covariance technique indicate a clear dependence of carbon dioxide (CO_2_) exchange on the surface cover, with the tower surroundings acting as an overall source of carbon (C), except the more vegetated sector during the summer [22,23]. Urban CO_2_ budget studies in this city and other climate regions reveal a clear decrease of CO_2_ exchange with increasing fraction of natural area, as a result of a combination of factors that include the photosynthetic uptake, pointing to 80% as the natural fraction needed for a city to reach C-neutrality [24].

The provision of regulating ES varies intensively across cities, but also between the urban core and its hinterland [25], revealing important mismatches in ES supply and demand [26]. The comparison of more than 300 urban European areas highlights Sweden and Finland for their potential to provide above-ground C storage and evapotranspiration (a proxy for urban heat regulation capacity), as a result of the high proportion of urban forest, the lower population density and the inclusion of UGI in urban planning [25]. These results indicate that compact cities, proposed as a solution for reducing urban greenhouse gases (GHG) emissions, are not necessarily optimal for the provision of regulating ES. A study in Espoo, Finland, concluded that cities with a large fraction of single-family houses having gardens with trees have the largest C sink potential [27]. This type of cities will, however, lead to longer transport distances and thus increased GHG emissions if CO_2_-neutral transport services are not utilized.

Meteorological observations conducted on street trees in Gothenburg during summer reveal an average conversion of 30% (amounting to 206 W m^−2^) of midday’s incoming solar radiation into latent heat flux due to tree transpiration [28]. Despite this energy loss, the authors concluded that a cooling effect of tree transpiration was not observed during the day and that its intensity varied greatly with species and location. In the same city, the cooling capacity of urban nature, the so-called Park Cool Island (PCI), was quantified as 0.81 °C during daytime in the warm season [29], similarly to the average of 0.94 °C reviewed by Bowler et al. [30] for different climatic regions. The strongest cooling (around 1.5 °C) was observed on the hottest periods, stressing the potential relevance of this effect during heat waves and in a warmer future climate. However, this maximum PCI intensity is clearly lower than earlier observations conducted in Sweden (cf. [5]).

Notwithstanding the indications of a limited effect of leaf transpiration rate on daytime warming rates or air temperature [28], heat stress can be significantly reduced by street trees shading [31]. In clear and calm summer days, such cooling effect is well captured by detailed maps of mean radiant temperature (T_mrt_) simulated with the radiation model Solweig [32,33,34,35]. During heat-stress conditions, T_mrt_ is shown to decline almost linearly as a function of increasing vegetation cover [35]. Depending on the location and size of trees, average T_mrt_ can drop by up to 30 °C, reducing the number of hours per year with severe heat conditions (T_mrt_ above 60 °C) by 40 [36].

From a climate-sensitive planning and design perspective, some studies [31,34,36] conclude that deciduous trees should be preferred over evergreen at high latitude cities, aiming at reducing the blockage of solar radiation in wintertime. Because even defoliated trees present relatively low transmissivity of direct solar radiation (40 to 52% in the study by [31]), a “mosaic” of outdoor urban spaces is preferable. Such combination of shaded/sunlit areas and distinct ventilation patterns within short walk distances will ultimately enhance and prolong the use of outdoor spaces [33,36].

#### 3.2.2. Water Runoff Regulation

The literature shows that blue-green solutions are used in the Nordic countries to retain stormwater and surface runoff in the example of the Copenhagen’s cloudburst management plan, where Sustainable Urban Drainage Systems (SUDS), such as parks and playgrounds, can be flooded during heavy rainfall, while serving as recreational spaces in dry weather [37]. The integration of open drainage basins in urban recreational areas is likely a better adaptation strategy than larger sewer pipes or local infiltration units [38]. In addition, green spaces provide monetarily-measurable ES related to urban runoff management, a value that increases when benefits from improved water quality are added [39].

A popular case study is the Augustenborg neighborhood, located in Malmö (Sweden), where runoff was disconnected from the combined sewer systems in the late 1990s due to frequent basement flooding. Modelling of a cloudburst registered in 2014 shows 70% less flooded area after retrofitting with a blue-green stormwater system, which contributed to a controlled flooding. Peak flows in the surrounding pipe-systems were reduced by approximately 80% and levelled out the runoff [40]. In Copenhagen, estimates indicate that 60% of a 15 km^2^ sewer catchment could be disconnected using SUDS that include green roofs, rain gardens, bioswales, soakaways, or wet/dry basins, thereby reducing the combined sewer overflows to the local stream [41]. However, from the comparison of adaptation strategies in Copenhagen and Beijing regarding flood control, an unbalance between conventional and nature-based solutions was identified as a result of political pressure for fast results, a lack of suitable green spaces, and insufficient experience with these alternative solutions [42].

Street trees transpiration is also a potentially relevant factor in urban stormwater management. A 4-year-long study in Helsinki [43] indicated that the average water use of *Alnus glutinosa* f. *pyramidalis* trees exceeded 50% of the precipitation with only 20% canopy cover, while for *Tilia* × *vulgaris* a cover of 60–70% was necessary to reach such rate.

Several of the reviewed papers deal with the hydrological performance of green roofs. Such technical solutions have integrated the urban stormwater management in Nordic countries with the purpose of reducing runoff volume and peak flows, avoiding sewage pipe system overflow. Annual runoff volume from three extensive *Sedum* roofs in Odense and Copenhagen, Denmark, was estimated as 43–68% of the total annual precipitation, with the thickest substrate and drainage layer (6–8 cm) having the best performance in peak flow reduction [44]. Peak time delay was found to vary greatly depending on rainfall intensity, with the lowest delay observed for the rainfall events with the highest return period.

In a comparison of green roofs performance in Norway, Sweden, Iceland and the UK [45], the warmest and driest locations reached the highest retention of stormwater, 58% of the annual precipitation, against 17% in the coldest and wettest regions. However, the wettest places attained the highest annual retention in absolute value. It is also worth noting that all locations showed a considerable retention of stormwater during the summer, ranging from 52 to 91%.

#### 3.2.3. Air Quality Regulation

The effect of urban vegetation on air quality was investigated at a number of sites in Lahti and Helsinki, Finland [46,47], each site consisting of a pair of sampling units located in a tree covered area and in an adjacent open area. No significant differences between the two sites were detected in the concentration of nitrogen dioxide (NO_2_) or anthropogenic volatile organic compounds (VOCs). For particulate matter (PM), however, the deposition fluxes (a proxy for PM10 concentration) were significantly lower in the tree-covered sites, suggesting that the UGI is effective in reducing PM10 concentrations. The difference in concentrations between tree covered and open sites was similar in summer and winter indicating that the possible improvement of air quality was not caused by the trees’ foliage. Higher concentrations of all investigated polycyclic aromatic hydrocarbons (PAHs) were found at the tree covered sites during summer. During winter, some of the PAHs displayed a contrasting pattern, with significantly lower concentrations at the tree-covered sites. Another study [48] confirmed that there were no significant differences in the concentration of gaseous pollutants, namely NO_2_, ozone (O_3_) and VOCs, between vegetated and open areas at identical distances from a road in the Helsinki metropolitan area, while PM deposition (interpreted as coarse PM concentrations) was significantly lower at the vegetated sites. However, none of the observed vegetation properties (canopy closure, tree number and size, ground vegetation) correlated significantly with the magnitude of the difference between PM deposition at vegetated and open locations, hence the mechanism behind PM uptake by UGI in Nordic cities remains partly unresolved. Clearly enhanced concentrations of NO_2_ were found both in front and inside green belts compared to concentrations at the same distance from the road but in open areas [49]. It was not possible to discern any effect on the NO_2_ concentrations behind the green belts. These findings were observed both during winter and summer. These studies refute the hypothesis that UGI, mostly in the form of deciduous trees, reduces local concentrations of gaseous pollutants in Finnish cities.

In contrast, significantly lower NO_2_ (and particle-bound PAH concentrations) were reported at a vegetated site compared to an adjacent, tree-less, site at similar distance from a busy road in Gothenburg, Sweden [50]. Lower NO_2_ were also reported inside a forest patch in Gothenburg compared to a co-located open site [51]. The vegetated site was, however, located slightly further from the major road compared to the open site. The authors [51] noted that the impact on the NO_2_ differences between the two sites did not vary systematically over time, although the sampling largely covered the period of leaf senescence. As reported both in [51] and [50], no effect on local O_3_ concentrations could be detected from stands of broadleaved trees in Gothenburg.

The tree species had different efficiencies with regards to PM uptake in a nursery along a busy motorway outside Stavanger, Norway [52]. Pine (*Pinus* spp.) and birch (*Betula pendula*) were among those with particularly high accumulation, although particles of different sizes were taken up with distinct efficiency depending on tree species. Similar methods were used to compare the efficiency of PM uptake in two species of coniferous trees grown along a highway outside Stavanger, Norway [53]. By comparing the PM content on needles with different age they show that there is a cumulative deposition in the 2-year lifetime of the needles.

#### 3.2.4. Noise Regulation

Measurements in a large park in Gothenburg revealed that noise levels were reduced by the emergence of leaves on trees. The results confirm that noise attenuation increases with distance, and when more greenery separates the walker from the road [50]. Reduced noise can have substantial health benefits, including the improvement of concentration problems and better sleep quality when the dwelling´s windows face a green space in Malmö [54], or beneficial short-term changes in cardiovascular risk factors among visitors of urban parks in Helsinki [55].

The diversity of relaxing nature sounds have also been associated with benefits to well-being and health [56,57]. The perception of soundscape in an urban park is also closely linked to how people perceive its suitability for everyday recreation and psychological restoration [58,59,60]. Research suggests that high noise levels, particularly from traffic, are associated with a low probability of restoration in small urban parks and should be avoided in the design of such infrastructures [61,62].

#### 3.2.5. Promotion of Well-Being and Health

The well-being and health of urban dwellers are not only intrinsically interconnected, but are also closely linked to the quality of the environment, of which the accessibility and use of green areas play an important role. Despite indications that the use of green spaces increases with the residence proximity (e.g., [63]), and a general recommendation of maximum 300 m (approximately 5 min walk) from home for the everyday use of recreational areas [64,65,66,67], there is no evidence-based consensus on a maximum distance that enables benefits to health [68], in line with findings from a review of accessibility metrics [69].

Similarly, there is an apparent lack of agreement relating the optimal recreation size of urban green spaces [68], with physical activity levels potentially increasing with park size [70], but also small parks as potentiating social cohesion and psychological restoration [71,72].

In fact, an analysis of 72 parks in Oslo, Stockholm and Copenhagen, reveals that, although bigger parks are more likely to offer possibilities for restoration, some of the smallest parks attain the highest restorative value ratings [71]. Small public urban green spaces (SPUGS) such as “pocket parks”, with maximum size between 3000 [71] and 5000 m^2^ [73], may satisfy the need for everyday outdoor experiences and promote restoration in dense urban areas, in the example of the “pocket park programme” of Copenhagen [62]. Given the competition for space in compact cities, green roofs may also offer psychological benefits for people and habitats for a number of species [74]. Further, the UGI concept embodies an interconnected network of green spaces [75]. Ecological (or green) corridors are essential for maintaining interconnected habitats for species and thus biological diversity, while serving human movements and, therefore, creating recreational experience opportunities, as shown in Kuopio in Finland [76], Kristianstad in Sweden, and Copenhagen in Denmark [77].

A few indicators or measures of urban nature availability or accessibility have been developed and/or applied in a Nordic context, including the urban green space indicator (UGSI) [68] and the walkability index [78], but also combining people´s experiences and perception of environmental quality through Public Participatory Geographical Information System (PPGIS) methods [66,67,79,80,81]. The frequency and purpose of using green spaces does not depend on simply proximity and size, as indicated by studies in Norway [82], Denmark [64,83] and Finland [84], pointing to additional factors. A calm environment (e.g., [85]), sounds of nature (e.g., [86]), aesthetics (e.g., [87]), shading and cooling [67] and, in general, the contact with nature (e.g., [66]) are values that people look for when visiting a green area near home.

ES valuation indicates that aesthetic and recreational benefits have the highest economic value of all ES [21,88,89]. Such values are, however, perceived in different and subjective ways. Women and elderly can be more sensitive to aesthetics [56] and express greater calmness when hearing nature sounds [90]. Additionally, self-rated “nature-oriented” individuals were shown to score higher the aesthetics and nature sounds than “urban-oriented” [87]. The aesthetics feature of greenery seem to be also a stimulating factor for bicycle commuting in inner Stockholm, indicating a positive relation with physical activity [59].

In overall, the experiences provided by the contact with UGI have positive impacts on emotional and physical well-being, sleep quality and perception of general health [91]. The most restorative environments for stressed individuals have been characterized by a combination of “refuge”, “nature”, “rich in species” and a low presence or absence of a “social” dimension (the latter, interpreted as an environment that is equipped for social activities) [85]. Gardening with social interaction are, on the other hand, pointed out as having an important role on stress disorder therapy [92] and contributing to higher quality of life, especially for elderly [93].

The likelihood of psychological restoration has been shown to increase with the number of street trees and the presence of flower beds in Iceland [94], while sitting in an urban park was scored as more likely to support restoration in Sweden, over sitting in a café, shopping in a mall, or walking along a busy road [95]. Findings from a self-report study in Helsinki and Tampere, Finland, suggest that restorative experiences in favorite places near home, including green areas, may be linked to work-related worries [96]. However, despite positive effects on perceived stress relief caused by short-term visits to nature areas, no significant differences in the levels of salivary cortisol, a widely utilized stress marker, were observed in Helsinki [97]. In addition, conclusions from a 8-year follow-up survey indicate that living far from usable green areas in Finnish cities may increase the risk of overweight and obesity, which the authors associated with other pathways than physical activity, such as stress releasing [98].

### 3.3. Disservices

Green areas have been identified as a significant source of birch and grass pollen, in the example of Danish cities [99,100], which is considered a major environmental disservice of UGI (e.g., [3,6]). In addition, grass pollen distribution is shown to be particularly heterogeneous, with very high ground-level concentrations near unmanaged grass areas, adding a higher level of complexity to the study and understanding of these processes. In this scope, a land use regression study of the Helsinki metropolitan area suggests that grass pollen concentrations can be estimated with reasonable accuracy using geospatial data variables [101].

In the Swedish BAMSE birth cohort, greenness in a 500 m buffer zone from home was positively associated with allergic rhinitis during childhood (6–8 years) and early adolescence (10–12 years), and also with aeroallergen sensitization in the younger group [102]. However, the same study found an inverse association for cohorts in Germany and the Netherlands, indicating a location dependent effect. In line with these findings, the analysis of cohorts from Finland and Estonia shows that the amount of forest and agricultural land within 2–5 km from home was inversely and significantly associated with the risk of atopic sensitization in children of 6 years of age and older [103]. These results indicate that environmental biodiversity affects the composition of the human skin microbiota, which in turn may protect against atopy and potentially against other chronic inflammatory disorders, and that early-life exposure to green environments is especially important in this context.

The leaching of nutrients from vegetated roofs has also been pointed out as a potential negative impact. Runoff water quality analysis on an extensive green roof (3 cm soil thickness) in Augustenborg associated the phosphorus release to the use of fertilizer and the composition of soil material [104]. However, when compared with concentrations in regular urban runoff, green roofs were similar or lower for both nutrients and heavy metals. Observations of the phosphorus and nitrogen concentrations in the runoff from green roofs in Helsinki and Lahti (Finland) showed that amending the soil with biochar retained nutrients after the maturation of the system, also offering higher water holding capacity than a crushed brick mixture [105].

Detailed assessments of the life cycle of planted urban trees in Helsinki showed that, due to high C loss from an artificial growth media, net C sequestration did not come about until after approximately 30 years of tree growth [106]. Consequently, and as a planning recommendation, green zones should be spacious enough for trees to reach their full size and thus maximize their C uptake potential [27]. In addition, intensively managed green areas can likely act as nitrous oxide (N_2_O) sources, as shown in Helsinki [107].

There are also relevant aspects for city planning and design to be considered, such as the loss of green space due to the installation of open drainage solutions [38], or the impairment of sunlight by vegetation in winter [31,34,36], since even leafless deciduous trees can block up to 60% of direct solar radiation [31].

## 4. Discussion on Knowledge Gaps

Our review concludes that trees, bushes and other vegetation in cities have the potential to both increase and decrease the levels of air pollution in Nordic cities, in line with other studies (see the reviews, e.g., [1,4,7]). These effects are coupled to the UGI’s impact on natural emissions, its ability to take up gases and particles from the atmosphere, and changes to the transport by the mean wind and turbulent mixing as a result of the physical obstruction of flow by the vegetation.

While some measurement studies (e.g., [50,51]) do report lower concentrations of NO_2_ inside forest patches compared to open areas at similar distance from major roads, most other studies in the Nordic cities (e.g., [47,48,49]) find opposite or no differences. The UGI´s ability to reduce the atmospheric concentrations of coarse PM in Nordic cities seems to be more certain [46,48,50], although the mechanism and pathway of PM removal from the atmosphere is yet to be fully established.

We found no studies specifically addressing emissions of biogenic VOCs (BVOCs) from trees in Nordic cities. BVOCs may contribute to PM [108] and additional build-up of near-surface O_3_. Studies of UGI and near-surface O_3_ in Nordic cities did, however, not indicate excess O_3_ concentrations in connection to UGI, so the problem of detrimental BVOC emissions in Nordic cities is probably minor.

The majority of the air quality works used “IVL-type” diffusion samplers for the gaseous compounds and other, similar, passive long-term averaging methods for the determination of PM concentrations. Although some recent studies (e.g. [109]) complemented the passive samplers with active high-resolution instruments, our view is that there is room for additional studies using different measurement techniques and studying different cities.

In hydrology studies, the lack of multi-year measurements has limited the possibility of drawing conclusions on the long-term performance of green roofs, nutrient leaching processes or the effect of biochar [105]. Johannessen et al. [45] emphasized that the understanding about *Sedum* performance in cold and wet climates is deficient, and therefore the direct transfer of knowledge from drier climates is not sufficient. Additionally, the need for cost-benefit analyses of technical solutions aimed at handling urban runoff may hinder practical implementation and management [38,41].

The observed average cooling capacity of Nordic urban parks [29] agrees with data from other climatic zones [30], but research is limited to a few sites in short-term monitoring campaigns. There is also a need for long-term flux measurements representative of the climate and morphology of Nordic cities. The increasing crowd-sourcing of observations (e.g., *Netatmo Weathermap* [110], *WeatherObservationsWebsite (WOW)* [111] or *Temperatur.Nu* [112]), combined with the development of artificial intelligence techniques (e.g., [113]), provides denser observation networks and enhanced data coverage. Additionally, high-resolution weather and climate modelling will foster a more detailed understanding of the interactions urban surface/atmosphere, but issues as the uncertainty in the simulation of evapotranspiration and latent heat flux (e.g., [114]) or the validity of core parameterizations describing street canyon processes in urban canopy models need to be overcome.

Extensive computation of T_mrt_, mostly over large cities in Sweden, has given insights on the impact of shading on outdoor heat stress, but this parameter provides an incomplete description of thermal comfort by neglecting other factors of the human heat balance [33,34], such as the convective cooling of wind. This calls for integrated heat stress indices (e.g., the Physiological Equivalent Temperature (PET) or the Universal Thermal Climate Index (UTCI)), which provide a more comprehensive understanding of the thermal interaction between the human body and its surroundings, to be further exploited at high latitude cities. Furthermore, thermal, emotional and perceptual assessments of a physical place may be intertwined with psychological and cultural processes [115], which the thermal indices above fail to capture, opening exciting opportunities for multidisciplinary and interdisciplinary research.

In the same perspective, while GIS and map-based indicators represent efficient planning tools for mapping and measuring the accessibility to urban recreational areas [58,116], efforts to complement these with social aspects, namely the people’s perceptions (e.g., relating the attractiveness of natural areas [81]), should be promoted. This requires moving beyond land-use classes, as defined by European datasets (e.g., the Urban Atlas), and toward tools capable of capturing more detailed aspects of land use and its relations to the supply of urban ES [117], including cultural ones.

Independently from the primary design purpose of a given UGI solution (e.g., flood control, the establishment of connectivity, or the promotion of leisure and biodiversity), the conception and implementation of such type of infrastructure should strive for the maximization of the co-benefits. These may include providing shade during heat stress events or damping noise in city centers with heavy traffic. In parallel, there is a need to account for undesired side effects, namely by ensuring ventilation conditions that counteract eventual build-up of air pollution hot spots in street canyons, or by selecting adequate tree species capable of, e.g., providing good shading coverage in summer while allowing radiation to penetrate in winter and have low pollen emission.

In our review of the scientific literature, we found a lack of comprehensive and knowledge-based guidelines or recommendations for the planning and design of UGI in Nordic cities, despite these being proposed in grey literature (see, e.g., [15]), a lack of integration also identified by Brink et al. [8]. In practice, site specific cases call for tailored solutions that account for local conditions and allow for the optimization of ES provision while minimizing the above-mentioned disservices, but also possible conflicting effects. Several examples can be referred: (1) While planting trees is widely proposed as an effective measure to reduce radiant heat load (e.g., [36]) and promote well-being (e.g., [57]), it may conflict with limited space in densely built-up areas and narrow streets. (2) SPUGS offer enhanced well-being in dense cities, but the restoration potential may be compromised by disturbing surroundings, namely traffic noise [61,118], and limited enclosure, which restrict the psychological distancing from daily routines [94,119]. (3) While, on the one hand, large deciduous trees are preferred for reducing heat stress [34,36], pine trees, on the other hand, seem particularly effective in taking up atmospheric PM [52]. (4) Additionally, unmanaged grass areas favor the accumulation of soil organic C [27], but are a much larger pollen source than regularly mowed lawns [100]. Intensively managed vegetation has also been shown to act as a source of GHG, such as N_2_O and CO_2_ [106,107].

Generic approaches for the classification and evaluation of the ES of UGI have been proposed that account also for potential disservices (e.g., [6]), but negative effects are often neglected in ES valuation studies, to a great extent due to limited understanding of the underlying processes and resulting impacts. Further, only a few works [21,67,117,120] have conducted an integrated assessment of both regulating and cultural ES, which limits the full understanding of the processes and effects involved.

## 5. Conclusions

Our review addresses two major families of ES, regulating and cultural, because of their relevance in urban environments. Having in mind the scope of the analysis and methodological limitations, we conclude that despite the importance of flood regulating solutions in the Nordic countries, this topic has not dominated the scientific production in comparison to the other ES analyzed. Our contact with stakeholders shows, however, that this may not reflect the abundance of grey literature produced on this subject by e.g., municipalities. The results confirm that other hazards have triggered relevant research efforts. Local climate, and heat attenuation in particular, have driven substantial research in the period covered by the review. Although heat stress studies, and contrarily to urban flooding, have not been driven by past extreme events motivating an end-user demand, the awareness of the recent impacts in Sweden of the 2018 heat wave, and the increased likelihood of similar events in a future climate, will expectably foster the research on the potential of UGI for climate change adaptation in Northern Europe, in particular during heat waves.

In our assessment of the scientific evidence, we found diverging and seemingly inconsistent findings, especially in what concerns air quality. Despite the fact that the UGI´s ability to reduce the atmospheric concentrations of coarse PM seems more certain, we did not find support for the general assumption that UGI is unequivocally reducing the levels of air pollution in Nordic cities, which calls for additional research. There are also gaps in the understanding of long-term performance and function of green roofs in cold and wet climates that may create obstacles for practitioners.

Since densification, and the competition over land resources, can potentially compromise the provision of ES, small interconnected green spaces may promote, notably in more compact cities, the frequent use of green spaces that enables both psychological (through stress restoration) and physiological health (via physical activity). At the same time, these should provide a network of natural commuting links that offer cooling (by shading) and good air quality (by ventilation). In overall, this planning goal clearly states the close relationships and interactions among different ES provided by urban greenery, either regulating and cultural. From an urban planning and development perspective, we identify also a need of accounting for the intense seasonal changes in temperature and radiation, which stress the importance at this latitude of adopting a year-round strategy, translated into what is commonly known as climate-sensitive planning. Due to the implications of local context, no universal rules can be established for such practices, in agreement with Oke et al. [18].

Finally, the design of UGI, including the selection and arrangement of plants, should be guided by the assessment of local conditions and vegetation’s specific traits. It should also balance both the provision of ES and their side effects and disservices, aspects that seem to have been only marginally investigated, yet being fundamental for a full understanding of the role and performance of UGI in this region. We conclude that the difficulty in valuing trade-offs and side-effects will ultimately have practical implications and may compromise the implementation of UGI by municipalities. The scarce number of articles appraised explicitly involving end users or stakeholders points to a need of better engagement and communication.

Notwithstanding the intricacies and synergies between regulating and cultural ES, few studies in our selection accomplished, or have even attempted, an integrated evaluation. We argue that promoting interdisciplinary studies is fundamental to attain a holistic understanding of how plant traits affect the resulting ES; of the synergies between biophysical, physiological and psychological processes; and of the potential disservices of UGI.

With this overall perspective, a debate on how to optimize the benefits of urban nature in Nordic cities under the challenges of urbanization and climate change, while accounting for regional and local specificities, should be further endorsed by the research community, practitioners and decision makers.

## Data Availability

No data available.

## Appendix A

**Table A1:** The search string takes the general form *A AND B AND C AND D*, where *A* stands for keywords characterizing the geographical location, *B* is the spatial unit, *C* the UGI type, and *D* the topic. Keywords are separated by OR in the string, and * is a wildcard character.

*A*	nord*, ”Northern Europe”, Scandinavia*, Swed*, Denmark, Danish, Finland, Finnish, Iceland*, Norw*, Stockholm, Gothenburg, Malmö, Copenhagen, Oslo, Helsinki, Helsingfors, Reykjavik
*B*	city, cities, *urb*, metropolitan, hous*, building, town, street, wall, façade, roof, surface, catchment
*C*	vegetati*, plant, green*, “green infrastructure”, UGI, “green urban infrastructure”, “nature-based solutions”, NbS, “ecosystem services”, “ecosystem-based adaptation”, EbA, “urban forest”, “urban woodland”, park, garden, tree, courtyard, farm, lawn, leaf, leaves, foliage, canopy, Betula, Birch, Tilia, Linden, Lime, Acer, Maple, Platanus, Plane, Quercus, Oak, Aesculus, “Horse chestnut”, Fraxinus, Ash, Pinus, Pine, Prunus, Cherry, Populus, Poplar, Ulmus, Elm, grass, Sedum
*D*	health, wellbeing, well-being, comfort, climate, adapt*, heat, UHI, temperature, thermal, shad*, cool, PCI, evaporation, *transpiration, wind, ventilation, turbulen*, noise, flux, mixing, *water, flood*, rain*, precipitation, cloudburst, runoff, drain*, detain, percolation, infiltration, “air quality”, “air pollution”, particle, “particulate matter”, PM, PM10, PM2.5, BPM, “organic compound”, *VOC, isoprene, monoterpene, “carbon dioxide”, CO_2_, “carbon monoxide”, ozone, O_3_, “nitrogen oxides”, NO_x_, “nitrogen monoxide”, “nitric oxide”, “nitrogen dioxide”, NO_2_, “nitrous oxide”, N_2_O, “sulfur dioxide”, SO_2_, methane, CH_4_, aerosol, carbon, pollen, allergen*

**Table A2:** Database fields (“Nd” stands for undetermined or unspecified).

Fields	Options
Country	Nd (e.g., Northern Europe or Scandinavia); Denmark; Finland; Iceland; Norway; Sweden
City or region	(text entry)
Geographical scale	Nd; building; neighborhood; city; region; country
Spatial resolution (applies essentially to modelling)	Nd; <1 m; 1–10 m; 11–100 m; 101–1000 m; 1–10 km; >10 km
Timescale	Nd; <24 h (very short term); 1–6 days (short term); 1–3 weeks; 1–3 months; 4–6 months (seasonal); 7–12 months; >1 year (long term); >10 years (decadal)
UGI type	Nd; street trees; urban park or forest; garden; courtyard; square; lawn; green-roof; green-wall; urban farm; other
Topic	Nd; local climate (including heat); global climate; air quality; water runoff (including flooding); noise; health; well-being; pollen; other
Quantitative method	None; field campaign; existing observation network; satellite; wind tunnel; other experimental; local scale model; mesoscale model; statistical model or analysis; other
Qualitative method	None; survey/questionnaire/interview; any other form of meeting; other
Identified limitations or obstacles	(text entry)
Identified knowledge gaps	(text entry)
Identified future projections	None; climate projections; emissions projections; urban planning scenarios; other
Effect/impact quantification	(text entry)
Identified disservices	(text entry)
Identified climate adaptation solutions	(text entry)
End users or stakeholders involved/addressed	(text entry)
Identified planning recommendations	(text entry)

**Table A3:** Classification of papers reviewed per topic (shaded cells).

	Local Climate	Global Climate	Air Quality	Water Runoff	Noise	Well-Being	Psychol. Health	Physiol. Health	Pollen
[28,29,31,32,33,34,35,36,114]									
[23,24,27,89,106,107]									
[46,47,48,49,51,52,53]									
[37,38,39,40,41,42,44,45,104,105]									
[60]									
[61,63,64,65,66,68,70,73,77,78,81,84,87,93,116]									
[83,85,90,94,95,96,118,119]									
[99,100,101]									
[22,25]									
[26]									
[21]									
[43]									
[117,120]									
[67]									
[115]									
[88]									
[50]									
[59]									
[58,62]									
[54,57]									
[55]									
[56,71,72,74,79,80,82,86,91]									
[98]									
[92,97]									
[102,103]									
